# Investments in implementation science are needed to address the harms associated with the sexualized use of substances among gay, bisexual and other men who have sex with men

**DOI:** 10.1002/jia2.25141

**Published:** 2018-06-05

**Authors:** Rod Knight

**Affiliations:** ^1^ British Columbia Centre on Substance Use Vancouver BC Canada; ^2^ Department of Medicine University of British Columbia Vancouver BC Canada

**Keywords:** drug use, men who have sex with men, public health, STD/STI

A growing body of epidemiological and behavioural research indicates that the use of both stimulants (e.g. cocaine, crystal methamphetamine) and depressants (e.g. alcohol, gamma‐hydroxybutyrate—GHB), used alone or in combination, are among the primary contemporary drivers of HIV and other sexually transmitted and blood‐borne infections (STBBI) experienced by some populations of gay, bisexual and other men who have sex with men (gbMSM) 1, 2, 3. For example, elevated rates of HIV and other sexually transmitted and blood‐borne infections (STBBI) among gbMSM are highly associated with the *sexualized use of substances,* that is, intensive polysubstance use to maximize pleasure and sociability with sex partners—a practice colloquially known in North America as “*Party ‘n Play*” (or “PnP”) and “*Chemsex”* in other regions (e.g. Europe). In most settings, however, the majority of intervention responses to sexual‐ and drug‐related risks tend to give rise to two parallel approaches to intervention. With some notable exceptions in a few major urban centres (e.g. the 56 Dean Street Clinic in London, United Kingdom), the vast majority of sexual healthcare services have not tended to address the harms associated with substance use among gbMSM. Similarly, conventional substance use prevention, treatment and care services tend to do so with little regard for an individual's sexuality and/or sexual behaviour. The Five key implementation “gaps” that are currently hindering our efforts to adapt interventions to address the harms that are associated with the sexualized use of substances among gbMSM are described below.

Gap 1: The spectrum of substance use is diverse among gbMSM who experience drug‐ and sexual‐related harms

Community drug use patterns vary significantly over time, underscoring the importance of implementing services that maintain “nimble” and evidence‐informed adaptations that are responsive to the needs of today's gbMSM. Furthermore, while previous research has been helpful in identifying how some sub‐groups of gbMSM are more likely to use substances, little is known about how the social and cultural contexts of *sexualized substance use* enhances or detracts from gbMSM's ability to prevent or reduce harm. For instance, little is known about the treatment and care needs of substance‐dependent gbMSM *versus* those who display more episodic substance use patterns 4. While the association of specific substances to HIV/STBBI risk behaviour is better understood (e.g. binge alcohol use; meth; cocaine; GHB), less is known about how particular *configurations* of substance use (e.g. combinations of “recreational” and “sex” drugs; specific routes of administration, including injection, inhalation or insufflation) may enhance or reduce sexual‐ and drug‐related risk 5. New research is needed to better understand how these phenomena occur so that substance use and sexual healthcare services can be adapted to address the corresponding harms.

Gap 2: Life course perspectives are critically needed

There are limited understandings about how substance use patterns occur across the life course of gbMSM's lives, including how key transitional periods (e.g. sexual debut; “coming out;” employment transitions) coincide with or shape substance use trajectories. Monitoring the patterns of substance use that produce sexual‐ and drug‐related HIV risks among various population sub‐groups of gbMSM (e.g. those who are: street‐entrenched; sex workers; clinically addicted *vs*. episodic users) is needed to optimize implementation strategies for the right group of gbMSM at the right time.

Gap 3: Healthcare providers’ perspectives remain absent

There is limited research examining the acceptability, feasibility or experiences of healthcare providers in providing comprehensive care for gbMSM who use drugs. Healthcare providers specializing in the provision of sexual healthcare may require additional resources and training to initiate culturally competent discussions about substance use with gbMSM, as well as to be adequately trained in the different options available for those displaying substance use disorders (e.g. referral pathways in a given setting; pharmacological options). Likewise, substance use care providers will also benefit from opportunities to better understand how contexts of sexualized substance use are associated with a combination of sexual‐ and drug‐related harms among some groups of gbMSM. Better engaging healthcare providers will also provide opportunities to identify the actionable levers available in a given setting to improve care and health outcomes among gbMSM who use drugs.

Gap 4: We do not know what the best “mix” of interventions are in any given context

There are real shortcomings to our current approach to addressing *syndemics* among gbMSM, that is, interrelated health inequities (e.g. mental health issues, HIV/STBBIs, substance use disorders) that are produced and reinforced by structural inequities such as stigma and barriers to care. Nevertheless, public health policy and community‐based interventions that address the patterns of sexual‐ and drug‐related HIV risks experienced by gbMSM within the context of supporting comorbid health issues (e.g. mental health) may provide gbMSM with the support they need to engage with other intervention modalities, including clinic‐based services (e.g. engagement with specialized health promotion case managers) and/or uptake of pharmacological regimens (e.g. Pre‐Exposure Prophylaxis—PrEP; pharmacotherapies for substance use disorders).

Gap 5: A variety of ethical questions remain unanswered

There are a variety of ethical considerations that require careful attention as interventions are adapted to address drug‐ and sexual‐related harms experienced by gbMSM. For example, previous discourse in this area has challenged the public health impetus to intervene “on” gbMSM's sexual lives, a population who has experienced decades of public health intervention and surveillance. Integrating community‐based principles (e.g. sex positivity; anti‐oppression) with key team science principles (e.g. high‐quality communication; mutual trust; power sharing) may allow those working in this area to address the ethical challenges “head on.”

## 1 Conclusion

Filling the key implementation “gaps” identified above will provide new opportunities to effectively and ethically address the harms that are associated with the sexualized use of substances among gbMSM. As funding commitments are dedicated towards the growing field of implementation science (e.g. by: US National Institute on Drug Abuse; Canadian Institutes of Health Research), the onus will be on integrated and multi‐disciplinary teams (e.g. researchers, interventionists and community stakeholders) to collect, monitor and respond to “feedback” (i.e. adaptations) in ways that optimize (and effectively integrate) the substance use and sexual healthcare intervention “landscapes” for gbMSM.

## Competing interests

None to declare.

## Authors’ contributions

RK drafted and finalized the manuscript.
